# Insecticide treated nets use and its determinants among settlers of Southwest Ethiopia

**DOI:** 10.1186/s12889-016-2768-8

**Published:** 2016-02-01

**Authors:** Tsegaye Berkessa, D. Oljira, B. Tesfa

**Affiliations:** 1 Department of Public Health, Faculty of Public Health and Medical Sciences, Mettu University, Mettu, PO Box 318, Ilu Ababor Ethiopia; 2 Department of Midwifery, Faculty of Public Health and Medical Sciences, Mettu University, Mettu, PO Box 318, Ilu Ababor Ethiopia

**Keywords:** ITN utilization, Settlers, Malaria prevention, Ethiopia

## Abstract

**Background:**

Ethiopia is rapidly increasing insecticide-treated nets (ITNs) coverage to combat malaria, but adequate follow-up and factors affecting use of ITNs is lacking. The aim of this study was to assess determinants of the use of ITNs in a southwest area of Ethiopia.

**Methods:**

This cross-sectional survey was conducted in the Chewaka district settlement area of southwest Oromia from March to May, 2013. Kebeles were stratified by degree of urbanization (rural, peri-urban, or urban). Randomly selected households, which had been freely supplied with at least one ITN, were surveyed using a pre-tested, structured questionnaire administered through household interviews. Logistic regression analysis was used to examine the association between use of ITNs and determinant factors.

**Results:**

Of 574 households surveyed, 72.6 % possessed ITNs and 80 % of these had been used the night before the survey. The most common reasons for the absence ITNs in the household identified in this study were ITNs were old and therefore discarded and that households use ITNs for purposes other than their intended use. The multivariate analysis found that knowledge of malaria transmission by mosquito bites (Adjusted OR = 3.44, 95 % CI: 1.80–6.59), and washing of ITNs at least once by households (Adjusted OR = 2.66, 95 % CI: 1.35–5.26) were significantly associated with an ITN being used by households. The mean possession was 1.59 ITN per household (3.57 persons per an ITN). One hundred fifty four (36.9 %) of ITNs had at least one hole/tear. Among these, 108 (70.1 %) ITNs had at least one hole/tear with greater than 2 cm and 29 (18.8 %) had greater than seven holes/tears.

**Conclusions:**

This study in Southwest Ethiopia showed a high proportion of net ownership compared to a household survey from Ethiopia which included in the World Malaria Report. Despite somewhat high percentages ITN ownership, the study demonstrated there was still a gap between ownership and use of ITNs. Use of ITNs was affected by knowledge of malaria transmission by mosquito bite and washing of ITNs at least once by households. Intensive health education and community mobilization efforts should be employed to attempt to influence these factors that significantly affect ITN use.

## Background

Malaria remains a major public health problem particularly in sub-Saharan Africa. In 2012, malaria was responsible for over 1.1 million deaths globally [1] and was endemic in 104 countries with substantial geographic disparities. Around 81 % of the malaria incidence and 91 % of the malaria deaths in 2010 occurred in Africa [2]. Approximately 52 million people in Ethiopia (approximately 68 % of the national population) live in malaria risk areas, primarily at altitudes below 2000 m [3]. In Oromia 75 % of the land is considered malarious, accounting for over 17 million people at risk of infection [4].

Insecticide-treated bed nets (ITNs) are a means of malaria control and prevention [5]. The impact of ITNs on reducing malaria episodes is well documented [6, 7]. Use of ITNs is one of the major vector control measures in Ethiopia. More than 20 million ITNs were distributed between 2005 and 2007, enabling 68 % of the households living in malaria-endemic areas to own at least one ITN. Indeed, 15 million ITNs were distributed in 2010 and 2011 to replace long-lasting insecticidal nets (LLINs) distributed previously [3, 8]. Beyond household possession of ITNs, it is crucial to understand household use of ITNs. The ITNs that are available at a household level may be left unused; or alternatively, even if ITNs are used, usage may be intermittent and/or vulnerable members of the household may not be given priority. The maximum malaria reduction impact of ITNs will only be achieved if people acquire nets, treat/re-treat them, make sure that the most vulnerable household members sleep under them, and use nets through the year [9].

Studies in various African countries, including Ethiopia, have revealed discrepancies between ITN possession and use [8, 10–12]. Yet, there is no properly documented evidence regarding the coverage and use of ITNs among settlers in malaria-prone areas. This study was designed to investigate the possession, use, and factors affecting use of ITNs in the Chewaka district settlement area. This study also helped to evaluate the local ITN programs with reference to the Abuja targets.

## Methods

### Study setting and design

This cross sectional survey was conducted in Chewaka district settlement, located approximately 566 km from Addis Ababa in the Southwest Oromia Region of Ethiopia. It has a total population of 65,850, with people residing in 28 kebeles. The climatic condition of the area is a tropical zone referred to as ‘cola’. It accounts 54.22 km^2^ of land area which is all the land masses are suitable for settlement. The annual temperature and rainfall varies from 37 to 40°c and 1000 to 1200 mm respectively, and the altitude ranges from 900 to 1400 m above sea level. There is known the successful settlement in Oromia and by sesame seed production. The main rivers in the area include the Didesa and Dabana, which are tributaries of the Abay River. In the district there are three health centers, twenty seven health posts, eighteen clinics and one drug vendor (Chewaka district annual health office report, unpublished).

### Sample size and sampling technique

The sample size for the survey was calculated by using the formula for a single population proportion, including a 95 % CI, 5 % margin of error, and estimate of 73 % of household using ITNs based on a previous study in Ethiopia [13]. With a 5 % adjustment for non-response rate and a design effect of 2, the resulting calculation for a total sample size was 636 households. The study employed a multi-stage sampling technique, taking into consideration that socio-demographic factors affecting ITN use might differ based on the household’s distance from the district town. The 28 malarious kebeles of the district were first stratified into three groups (urban, semi-urban, and rural kebeles), and then six kebeles were randomly selected by lottery method by proportional allocation to size (1 from the first, 2 from the second and 3 from the third stratum). Health extension workers (HEWs) distributed ITNs to the community at their vicinity/households during free mass-distribution campaigns. Households were randomly selected from a list provided by the district administration

### Data collection method and analysis

Data were collected by diploma health professionals after training using a pretested, structured questionnaire prepared in English and then translated into the local language of Afan Oromo. The questionnaire was adopted from instruments developed by the Roll Back Malaria (RBM) partnership Monitoring and Evaluation Reference Group by the WHO and UNICEF [14]. Data collection was conducted during the peak malaria transmission period, from March to May, 2013. Data collectors administered the questionnaire through household visits. Information was primarily collected from the head of households (father or mother), or if this was not possible, from another adult household member. The condition of household nets was inspected by use of a checklist. Data was entered using Epidata version 3.1 and Stata version 11.0 was used for analysis. Descriptive statistics provided means and percentages related to socio-demographic characteristics; knowledge of malaria transmission and prevention; and possession, use, and condition of ITNs. Through logistic regression, adjusted odds ratios were calculated to identify predictors of ITN use.

### Ethical consideration

Ethical clearance was obtained from the Mettu University Faculty of Public Health and Medical Sciences Institutional Research Ethics Review Committee, as well as from the zonal and woreda-level health offices. Before each interview, researchers sought consent from each respondent.

## Results

### Socio demographic characteristics

A total of 574 households participated in this study with a response rate of 90.3 %, of which 377 (65.7 %) were heads of households. The majority of the respondents 352 (61.3 %) were female. The mean (SD) age of the respondents was 30.6 (8.9) years and the mean (SD) family size was 5.7 (2.2). Of the total households, 470 (81.9 %) had at least one child under 5 years of age (in total 703) and 86 (15 %) had pregnant women (one in each household). A majority of respondents (379 or 66 %) were illiterate (unable to read and write) and 414 (72.1 %) were farmers (Table 1).

**Table 1:** Socio-demographic characteristic of respondents in Chewaka district, South West Ethiopia, 2014

Variables (*n* = 574)	Frequency	Percent
Sex		
Male	222	38.7
Female	352	61.3
Age (years)		
15–29	286	49.8
30-44	237	41.3
≥ 45	51	8.9
Marital Status		
Single	50	8.7
Married	507	88.3
Divorced	10	1.8
Widowed	7	1.2
Educational level		
Illiterate	379	66
Literate	195	34
Occupation		
Student	31	5.4
House wife	70	12.2
Governmental Employee	19	3.3
Merchant	35	6.1
Farmer	414	72.1
Other	5	0.9
Monthly income of HH (birr)		
< 100	13	2.3
100–299	129	22.5
300–499	167	29.1
500–799	149	26
> 799	116	20.2
Family Size		
Less than three	35	6.1
Three to four	158	27.5
Five to six	179	31.2
Seven and above	202	35.2
Presence of radio in the HH		
No	293	51.1
Yes	281	48.9
Presence of television in the HH		
No	544	94.8
Yes	30	5.2

#### Knowledge of respondents about malaria transmission and prevention

From the total respondents, 342 (59.6 %) mentioned mosquitoes bite as the main mode of transmission for malaria. The other means of transmission reported included (232, 40.4 %): living near stagnant water (138, 24.0 %), feeling cold (16, 2.7 %), presence of waste (35, 6.1 %), drinking dirty water (24, 4.2 %), being hungry and being in the rain (both 9, 1.6 % respectively). The majority of respondents (208, 39.3 %) reported ITNs as the main preventive measure against malaria; followed by proper waste disposal (89,16.8 %), taking tablets (85,16.1 %), use of aerosol sprays (59,11.2 %), drainage of breeding sites (59,11.2 %), use of traditional remedies (19,3.6 %), and fumigation (9, 1.7 %). The majority (495, 86.2 %) of respondents had ever heard/seen messages about ITNs. Most (56598.4 %) of the respondents believed that sleeping under an ITN is beneficial, and only 42 (7.3 %) respondents reported problems associated with sleeping under an ITN (Table 2).

**Table 2:** Knowledge of respondents about the transmission mechanism and preventive measures of malaria, ITN awareness and associated factors in Chewaka district, South West Ethiopia, 2014

Variables	Frequency	Percent
Main transmission mechanism of malaria (*n* = 574)		
Bitten by mosquito	342	59.6
Living near collected water	138	24.0
Feeling cold	16	2.7
Presence of wastes	35	6.1
Drinking dirty water	24	4.2
Being hungry	9	1.6
Being in the rain	9	1.6
Other	1	0.2
Main preventive measures of malaria (*n* = 529)		
Use of ITN	208	39.3
Take tablet	85	16.1
Proper disposal of wastes	89	16.8
Use of traditional remedies	19	3.6
Fumigants	9	1.7
Use insecticide spray	59	11.2
Drainage	59	11.2
Others	1	0.2
Ever heard education messages about ITNs (*n* = 574)		
No	79	13.8
Yes	495	86.2
Think that sleeping under ITN have benefit (*n* = 574)		
No	9	1.6
Yes	565	98.4
The benefits of sleeping under ITN (*n* = 565)		
Don’t get bitten by mosquito	274	48.5
Don’t get bothered by other mosquito	95	16.8
Don’t get malaria	191	33.8
To get warmth	5	0.9
Believe that sleeping under ITN has problem (*n* = 574)		
No	532	92.7
Yes	42	7.3
Problems associated with sleeping under ITNs (*n* = 42)		
Difficult to get at night	18	42.9
It is too hot to sleep under ITNs	14	33.3
It takes time to tuck a net each night	5	11.9
Mosquito can still bite through ITN	4	9.5
No enough air when sleeping under	1	2.4

#### ITN possession and use

All households (*n* = 574) that participated in this study were freely provided with at least one ITN by the district health office. Two hundred ninety seven (51.7 %) households were supplied with one, 215 (37.5 %) with two, 61 (10.6 %) with three ITNs and 1 (0.2) with four ITNs. In total, 914 ITNs were supplied to the households included in this study. Of these, 731 (80 %) were reported as being used by households. Use varied among strata at 75 %, 84.9 % and 82.7 % for rural, semi urban and urban strata respectively. Among ITNs that were used, about 37.4 % had at least one hole. Mean possession was 1.59 ITNs per household (3.57 people per an ITN).

ITNs were not found in 157 (27.4 %) households at the time of the survey. The reasons for absence of ITNs included: lost or stolen (24, 15.3 %), used for other purposes (e.g. storage of sorghum, to make fences, protect bulls from insect bites) (46, 29.3 %), and thrown away as old (84, 53.5 %), and gave to others (3, 1.9 %).In addition, 20 (4.8 %) of the households did not use their nets due to: hot to sleep under the net (7, 35 %), housing construction problems (lack of appropriate place for hanging the net) (6, 30 %), absence of mosquitoes (2, 10 %), fear of its toxicity (2, 10 %), absence of bed (2, 10 %), and perception that ITN could not prevent malaria (1, 5 %). This implies that 334 (80 %) of the households used at least one of their freely supplied ITNs. The night before the day of the survey, 77.4 % (418) of all children under five and 75 % (54) of all pregnant women slept under an ITN (Table 3).

**Table 3:** ITN possession and utilization by households in Chewaka district, South West Ethiopia, 2014

Variables	Frequency	Percent
Number of ITNs freely supplied for HHs		
One/HH	297	51.7
Two/HH	215	37.5
Three/HH	61	10.6
Four/HH	1	0.2
Utilization of ITNs by HHs		
Currently used	334	80.1
Not used	83	19.9
Availability of at least one freely supplied ITNs		
No	157	27.4
Yes	417	72.6
Reason for unavailability		
Lost/stolen	24	15.3
Used for other purpose	46	29.3
Old then thrown away	84	53.5
Given to others	3	1.9
Households reported use at least one of their available ITNs		
No	20	4.8
Yes	397	95.2
Frequency of using their ITNs		
Consistently throughout the year	278	70
Intermittently	119	30
Time they use intermittently (*n* = 119)		
During rain	92	77.3
After rain	7	5.9
As they like	2	1.7
When hearing mosquito buzzing	18	15.1
Reason for not using the available ITNs (*n* = 20)		
Absence of mosquito	2	10
Absence of bed	2	10
ITNs do not prevent malaria	1	5
Afraid of its toxicity	2	10
ITNs too hot to sleep under it	7	35
Housing structure affects ITN use	6	30
Children under 5 years age slept under ITN in previous night (*n* = 540)		
No	122	22.6
Yes	418	77.4
Pregnant women slept under ITNs previous night (72)		
No	18	25
Yes	54	75

#### The condition of ITNs

Among households who owned ITNs (*n* = 417) at the time of the survey, 305 (73.1 %) had been washed at least once and100 (24 %) had been washed three or more times. One hundred fifty four (36.9 %) ITNs had at least one hole/tear. Among these, 29 (18.8 %) ITNs had greater than seven holes/tears and 108 (70.1 %) ITNs had at least one hole/tear greater than 2 cm (Table 4).

**Table 4:** Condition of ITNs in households who owned ITNs, in Chewaka district, South West Ethiopia, 2014

Variables	Frequency	Percent
Age of ITNs (*n* = 417)		
< 1 year	188	45.1
1–2 years	126	30.2
>3 years	103	24.7
Shape (*n* = 417)		
Rectangular	413	99
Conical	4	1
Color (*n* = 417)		
White	4	1
Green	143	34.3
Blue	270	64.7
ITNs ever been washed (*n* = 417)		
Yes	305	73.1
No	112	26.9
Frequency of washing (*n* = 305)		
One to three times	232	76.1
Four to six times	50	16.4
Seven or more times	23	7.5
Presence of hole/tear on ITN (*n* = 417)		
Yes	154	36.9
No	263	63.1
Number of holes/tears (*n* = 154)		
1-7	125	81.2
>7	29	18.8
Size of holes/tears (*n* = 154)		
≤ 2 cm	46	29.9
> 2 cm	108	70.1

#### Determinants of ITN utilization

Factors associated with use of at least one ITN by households were knowledge of malaria transmission by mosquito bite (Adjusted OR = 3.44, 95 % CI: 1.80–6.59), and ITNs washed at least once by household (Adjusted OR = 2.66, 95 % CI: 1.35–5.26). Sex and age of respondents, number of ITNs freely supplied, presence of children under five / any children in the household, and age of ITNs were not associated with the use of ITNs by households, when use was adjusted for the other factors (Table 5).

**Table 5:** Final logistic regression model for household’s ITNs use in Chewaka settlement, South West Ethiopia, 2014

Variables	ITN Utilization Status		Crude OR (95 % CI)	Adjust OR (95 %) CI
	No	Yes		
Sex				
Female	61	196	1.00	1.00
Male	22	138	1.95 (1.14–3.33)	1.60 (0.87–2.94)
Age (years)				
15–29	31	175	1.00	1.00
30–44	40	136	0.60 (0.36–1.01)	0.59 (0.32–1.07)
≥ 45	12	23	0.33 (0.15–0.75)	0.54 (0.20–1.42)
Knowledge of malaria transmission by mosquito bites				
No	35	40	1.00	1.00
Yes	48	294	5.36 (3.10–9.26)	3.44 (1.80–6.59)^*^
Number of ITNs freely supplied for HHs				
One/HH	30	174	1.00	1.00
Two/HH	38	131	0.59 (0.35–1.01)	0.77 (0.43–1.40)
Three and above/HH	15	29	0.33 (0.16–0.69)	0.80 (0.33–1.92)
Age of ITNs				
< 1 year	49	139	1.00	1.00
1-2 years	18	108	2.11 (1.16–3.84)	0.72 (0.33–1.57)
> 3 years	16	87	1.92 (1.03–3.45)	0.76 (0.33–1.74)
ITNs ever been washed				
No	42	70	1.00	1.00
Yes	41	264	3.86 (2.33–6.40)	2.66 (1.35–5.26) ^*^
Is under five child/ children in the HH				
No	18	41	1.00	1.00
Yes	65	293	1.98 (0.07–3.66)	1.35 (0.65–2.81)

* *P* < 0.05 - Significantly associated

## Discussion

The study showed that ownership and use of ITNs in the study area were 72.6 % and 80.1 % respectively. The percentage of children under 5 years of age and pregnant women not using ITNs exceeded that of other adults. The two factors strongly associated with use of ITNs the night before the survey were knowledge of malaria transmission by mosquito bites and ITNs being washed at least once by households.

The proportion of households possessing ITNs was higher in this study compared to the average figure indicated in World Malaria Report of 2011 (median = 56 %) from household survey results [15]. This implies that the net distribution program is going well when compared to the Roll Back Malaria [16] and World Health Assembly targets [17]. In this study, use of ITNs was in line with WHO recommendation of 80 % utilization [15]. High rates of use achieved within a short period of time demonstrates acceptance of nets by users as a major malaria control tool and reflects the concerted efforts of the Ministry of Health. However, more than half of households had just a single net, and on average four individuals shared a single net. This issue requires great attention, because the national policy aims to provide one ITN for every sleeping space (approximately one net per 1.8 persons in malaria-endemic areas <2000 m) [15]. Hence, to attain sustainable control of the disease, households in the study area require extra nets to reduce the occupant per net gap.

The mode of malaria transmission identified by 59.6 % of respondents in this study was mosquito bites. This result is higher than the findings from a study in Wonago Woreda, Southern Ethiopia, where 42.3 % of respondents also listed mosquito bites [18]. But, less compared to a study done in Oromia and Amhara regional state, Ethiopia (67.9 %) [19]. Respondents’ perception of net use as a main preventive measure for malaria was also lower compared to a survey done in Southern Ethiopia [18]. The reason for this may be that the majority of respondents included in our study were from rural areas and had less access to health information. Nonetheless, about three quarters of our respondents had ever heard educational messages about mosquito nets, which is high compared with national survey results (41.0 %) [20]. This may be because our study included only households who were freely supplied with ITNs, which would contribute to increased exposure to educational messages. The difference might also be due to the presence of extensive promotion of ITNs currently underway in the country.

Presence of holes/tears on nets was also associated with malaria infection. Around 79 % of ITNs had holes/tears ≥ 2 cm, which is higher than a study done in Tanzania in which 45 % of ITNs had holes/tears ≥ 2 cm [21]. It is also higher than a survey conducted in Malawi, where 12.8 % of owners reported that nets had holes >2 cm [22]. The reasons for this may include structural issues in the house that pose challenges to hang nets, such that the nets are too short to fully cover sleeping areas, long duration in use of ITNs, and frequent washing on rough surfaces.

Our finding revealed that there was strong association between using ITNs and knowledge of malaria transmission by bite of mosquito, which is in line with a study done Ghana [23, 24]. Households with ITNs ever been washed shows significant increase of utilization compared to households with unwashed ITNs. This might be due to households having been encouraged to use clean ITNs as opposed to dirty ITNs.

About 20 % of ITNs were not used by households and 29 % of ITNs were used for other purposes such as storage of sorghum, to make fences, protect bulls from insect bites. This implies that distribution of nets to communities without health education on the importance of ITNs in prevention of malaria, as well as how to use nets, may not bring about the desired result. In this study education levels of respondents, and ever heard education messages about ITN were not associated with the use of ITNs. Age and sex of respondents were not significantly associated with ITN utilization in this study. Some studies reported similar findings [25, 26], while the others showed significant associations between these factors and ITN use [8, 27]. In addition, education levels and income did not significantly affect ITN use in our study which is in line with previous study done Ethiopia [8], Uganda [28] and Nigeria [29]. Given the cross-sectional nature of the results, interpretation of study results is limited. One of the major limitations of this study was that it relied on reported use of ITNs by households prior nights, without any means observation. Thus, the percentage of ITN use in this study might be overestimated due to self- reporting bias. In the future, research using a prospective cohort study design would be valuable.

## Conclusions

This study in Southwest Ethiopia showed a high proportion of net ownership compared to a household survey from Ethiopia which included in the World Malaria Report. Despite somewhat high percentages ITN ownership, the study demonstrated there was still a gap between ownership and use of ITNs. On average, four individuals shared a single ITN and over one third of ITNs had at least one hole/tear. The two factors strongly associated with ITN use in surveyed households included knowledge of mosquito bites as a main mode of malaria transmission, and washing of ITNs at least once. To achieve sustained control of malaria, household coverage of nets alone is not sufficient. Public health interventions should also address problems related to utilization and care of ITNs. Intensive health education and community mobilization efforts should be employed to influence the specific factors identified as affecting ITN use.

## References

[1] Lozano R, Naghavi M, Foreman K, Lim S, Shibuya K, Aboyans V (2012). Global and regional mortality from 235 causes of death for 20 age groups in 1990 and 2010: a systematic analysis for the Global Burden of Disease. Study 2010. Lancet.

[2] Nsungwa-Sabiiti J, Peterson S, Pariyo G, Ogwal-Okeng J, Petzold MG, Tomson G (2007). Home-based management of fever and malaria treatment practices in Uganda. Trans R Soc Trop Med Hyg.

[3] Federal Democratic Republic of Ethiopia. National malaria guidelines. Third edition. Ministry of Health. Addis Ababa, 2012.

[4] Alemu A, Abebe G, Tsegaye W, Golassa L. Climatic variables and malaria transmission dynamics in Jimma town, South West Ethiopia. Malar. J. 2011; 4(30). http://www.ncbi.nlm.nih.gov/pmc/articles 10.1186/1756-3305-4-30 PMC3055844 21366906

[5] WHO (2007). Insecticide-Treated Mosquito Nets: a WHO Position Statement.

[6] Lengeler C (2009). Insecticide-treated bed nets and curtains for preventing malaria, Cochrane Database of Systematic Reviews 2004.

[7] Eisele TP, Larsen D, Steketee RW (2010). Protective efficacy of interventions for preventing malaria mortality in children in plasmodium falciparum endemic areas. Int J Epidemiol.

[8] Loha E, Tefera K, Lindtjørn B (2013). Freely distributed bed-net use among Chano Mille residents, south Ethiopia: a longitudinal study. Malaria Journal.

[9] Net Mark research 1999–2006. Available at: http://www.netmarkafrica.org/research. (Accessed on: 15/08/ 2006).

[10] Macintyre K, Keating J, Okbaldt YB (2006). Rolling out insecticide treated nets in Eritrea: examining the determinants of possession and use in malarious zones during rainy seasons. Trop Med Int Health.

[11] Koreromp EL, Miller J, Cibulskis RE, Kabir Cham M, Alnwick D, Dye C (2003). Monitoring mosquito net coverage for malaria control in Africa: possession versus use by children under 5 years. Trop Med Int Health.

[12] Tsuyuoko R, Midizi SM, Dziva P, Makunike B (2002). The acceptability of insecticide treated mosquito nets among community members in Zimbabwe. Cent Afr J Med.

[13] Astatkie A, Feleke A (2009). Utilization of insecticide treated nets in Arbaminch Town and the malarious villages of Arbaminch Zuria District, Southern Ethiopia. Ethiop. J. Health Dev..

[14] Roll Back Malaria, MEASURE Evaluation, World Health Organization, and UNICEF. Guidelines For Core Population Coverage Indicators For Roll Back Malaria: To Be Obtained From Household Surveys, MEASURE Evaluation, Calverton, Md, USA, World Health Organization; 2006.

[15] WHO (2011). World Malaria Report.

[16] RBM. The African Summit on Roll Back Malaria, Abuja, Nigeria. WHO/CDS/RBM/200.17.RBM. Geneva: World Health Orginization; 2000, p. 556–6.

[17] World Health Assembly. Fifty-Eight World Health Assembly: Malaria Control. In vol Resolution 55/284. Geneva: World Health Organization; 2005

[18] Dagne G, Deressa W. Knowledge and utilization of insecticide treated mosquito nets among freely supplied households in Wonago Woreda, Southern Ethiopia. Geneva: Ethiop J Health Dev. 2008;22(1):34–41.

[19] Baume AC, Reithinger R, Woldehanna S (2009). Factors associated with use and non-use of mosquito nets owned in Oromia and Amhara Regional States, Ethiopia. Malaria Journal.

[20] Jima D, Tesfaye G, Deressa W, Woyessa A, Kebede D, Alamirew D (2005). Baseline survey for the implementation of ITNs for malaria control in Ethiopia. Ethiop J Health Dev.

[21] Erlanger T, Enayati A, Hemingway J, Mashinda H, Tami A, Lengeler C (2004). Field issues related to effectiveness of insecticide treated nets in Tanzania. Med. Vet. Entomol..

[22] Holtz TH, Marum LH, Mkandala C (2002). ITN use, anemia, and malaria parasitemia in Blantyre district, Malawi. Trop Med Int Health.

[23] Agyepong IA, Manderson L (1999). Mosquito avoidance and bed net use in the Greater Accra Region, Ghana. J. Biosoc. Sci..

[24] Binka FN, Adongo P (1997). Acceptability and use of insecticide impregnated bednets in northern Ghana. Trop. Med. Int. Health.

[25] Berie Y, Alemu K, Belay A, Gizaw Z (2013). Factors affecting utilization of Insecticide treated nets among people living with HIV/AIDs in Bahir Dar city, northwest Ethiopia. J Clin. Med..

[26] Atieli E, Guofa Zhou G, Afrane Y, Lee M, Mwanzo I, Githeko K (2011). Insecticide-treated net (ITN) ownership, usage, and malaria transmission in the highlands of western Kenya. Parasit Vectors.

[27] Aigbe W, Iwara O, Okongor E, Okino I (2012). Ownership and utilization of Insecticide Treated nets in Cross River States, Nigeria. J. Med. Sci.

[28] Sangare LR, Weiss NS, Brentlinger PE, Richardson BA, Staedke SG, Kiwuwa MS (2012). Determinants of use of insecticide treated nets for the prevention of malaria in pregnancy: Jinja, Uganda. PLoS ONE.

[29] Ye Y, Patton E, Kilian A, Dovey S, Eckert E (2012). Can universal insecticide-treated net campaigns achieve equity in coverage and use? The case of northern Nigeria. Malar. J..

